# Area per Player in Small-Sided Games to Estimate the External Load in Elite Youth Soccer Players

**DOI:** 10.5114/jhk/189421

**Published:** 2024-12-06

**Authors:** Vicente de Dios-Álvarez, Alexis Padrón-Cabo, Pello Alkain-Villa, Ezequiel Rey, Julen Castellano

**Affiliations:** 1 Faculty of Education and Sport Sciences, University of Vigo, Pontevedra, Spain.; 2 Faculty of Physical Activity and Sports Sciences, University of the Basque Country (UPV/EHU), Vitoria-Gasteiz, País Vasco, Spain.; 3 GIKAFIT research Group, University of the Basque Country (UPV/EHU), Vitoria-Gasteiz, Spain.

**Keywords:** performance, soccer training, locomotor activities, density

## Abstract

Small-sided games (SSGs) refer to game formats where there are variations in the pitch size, the number of players, and rules as compared to official soccer matches. The purpose of this study was to assess the relationship between GPS-derived external loads and the SSGs’ area per player (ApP) in elite youth soccer players to identify whether the ApP influenced GPS-derived external load measures to estimate external load variables from SSGs with different densities (i.e., ApP100: the ApP <150 m^2^•player^−1^; ApP200: the ApP ranged from 151 to 250; ApP300: the ApP > 251 m^2^•player^−1^). A cumulative count of 978 individual observations was undertaken, encompassing 15 diverse SSG configurations. The results showed moderate and large correlations between the ApP and external load measures for both U19 and U16 soccer players. The ApP300 induced higher locomotor activities than the ApP100 and the ApP200. However, the ApP100 showed higher mechanical (accelerations and decelerations) values compared to ApP200 and ApP300 conditions for both age groups. It was found that an ApP of less than 150 m^2^•player^−1^ in SSGs did not stimulate enough high-speed distance relative (HSDR), very high-speed distance relative (VHSDR) and sprint distance relative (SDR) compared to official matches in youth soccer players. However, the same ApP overestimated mechanical variables such as total high accelerations and decelerations relative to time (THACCR and THDECR, respectively). The findings of this study have the potential to facilitate the effective management of training loads tailored to specific fitness components of players.

## Introduction

Small-sided games (SSGs) refer to training exercises that differ from official soccer match conditions in terms of the pitch size, the number of players, and rules of the game (Sangnier et al., 2019). Currently, SSGs represent a widely employed approach in both adult and young soccer players due to their capacity to effectively engage various physical capacities along with technical and tactical skills (Sarmento et al., 2018), providing consequences in psychobiological responses during the games (Clemente et al., 2021; Clemente and Sarmento, 2020).

An accurate comparison between match and training drills (i.e., SSGs) seems to be necessary to plan the training session according to the requirements of the competition. This is of great importance and was highlighted by Castillo et al. (2021) who revealed that the match load consistently exceeded the training load. However, in line with Riboli et al. (2020), more research is needed to compare both locomotor and mechanical activities between SSGs and matches. Casamichana et al. (2012) compared SSGs with friendly matches and found that sprints in friendly matches were more frequent, of longer duration, and of greater distances compared to SSGs (3 vs. 3, 5 vs. 5 and 7 vs. 7). Nevertheless, in that research, the relative playing area for each player during SSGs was kept constant (210 m^2^•player^−1^). Previous scientific literature suggests that soccer and conditioning coaches can modify the characteristics of SSGs by manipulating various variables, including a training method (Branquinho et al., 2020), the pitch size, the number of players, exercise duration, the number of repetitions, and recovery time (Branquinho et al., 2021a) with the aim to mimic official match demands and optimize performance (Branquinho et al., 2021b).

The combination of two commonly used variables (Bujalance-Moreno et al., 2019), namely the number of players and the dimensions of the playing area, results in the concept of the area per player (ApP). This concept stands as a pivotal element within SSGs. Previous research has defined the ApP as the theoretical pitch area allocated to each player. It is determined by dividing the total pitch area (taking into account the length-to-width ratio) by the number of players present on the field (Casamichana and Castellano, 2010). From a practical perspective, the manipulation of the ApP during SSGs may help coaches regulate both locomotor activities and mechanical efforts. Overall, earlier studies have shown that larger playing areas result in an augmentation of metrics such as total distance covered (TDC), distance covered per minute, distance covered at various intensities, and the frequency of sprints (Casamichana and Castellano, 2010; Hodgson et al., 2014). Similarly, a study by Castillo et al. (2020a) showed that SSGs performed in large areas (196 m^2^•player^−1^) resulted in higher TDC, distance covered jogging, sprinting, the number of sprints, and maximal velocity, in comparison to SSGs played in smaller areas (98 m^2^•player^−1^). Previous research on youth soccer players also showed that increases in both the ApP and the number of players were associated with increases in TDC, player load (PL) and high-speed running distance (HSD) (> 16 km•h^−1^) (Castellano et al., 2015). However, it should be emphasized that those authors only analyzed three different types of the ApP (300, 200 and 100 m^2^•player^−1^). In this respect, recent investigations with professional soccer players and large amounts of data have shown that the ApP during SSGs was correlated with relative TDC, high-speed distance and sprint (Riboli et al., 2020; Sangnier et al., 2019). Hence, an increase in the ApP used could rise locomotor demands in soccer players. However, controversy exists regarding the effects of different types of the ApP on neuromuscular actions. In this respect, Riboli et al. (2022) showed inverse small correlations among accelerations and decelerations and the ApP across different ages categories in elite youth players. In contrast, a moderate association was identified between the ApP and the number of accelerations and decelerations in professional soccer players (Riboli et al., 2020).

Based on previous research, SSGs do not seem to stimulate high-intensity efforts and repeated sprints in the same way as soccer match play (Casamichana et al., 2012; Clemente et al., 2019; de Dios-Álvarez et al., 2024). The stimulus of SSGs alone could not be sufficient to stimulate locomotor demands of match-play (Pinheiro et al., 2022). Specifically, SSGs do not reproduce the distance covered at higher or very high-speed. However, those authors only analyzed SSGs during preseason with six different ApPs. Therefore, further research investigating the differences between official competition and SSGs played with different ApPs seems appropriate. In particular, HSD and sprint distance (SD) play a crucial role in soccer-specific performance enhancement and injury prevention (McCall et al., 2020; Mendiguchia et al., 2020). Consequently, it seems necessary to accurately quantify external load measures during SSGs aimed at physical development to assist coaches and conditioning professionals in optimizing performance (Sangnier et al., 2019).

Although there are a lot of studies analyzing the pitch size and the number of players involved during SSGs (Casamichana and Castellano, 2010; Casamichana et al., 2015), these studies only investigated a small number of possible ApPs (with a small number of observations). Therefore, knowing how the external load measures vary according to the ApP, with a large range of observations, could improve our understanding of the physical demands associated with SSGs (Praça et al., 2022). Nevertheless, to the best of our knowledge, only one study has addressed the relationships among many different ApPs and external load measures with young soccer players (Riboli et al., 2022).

A recent systematic and meta-analytical review highlighted the necessity of controlling the ApP among different SSGs formats to monitor the influence on external loads (Gibson-Praça et al., 2022). Therefore, the objectives of our study were twofold: (i) to evaluate the association between GPS-derived external loads and the ApP in elite youth soccer players, aiming to determine whether the ApP has an impact on external load measures, and (ii) to compare the physical demands of SSGs featuring different ApP values with those observed during official soccer matches.

## Methods

### Participants

The present study involved a total sample of 60 youth soccer players who competed at the highest possible level within their age category. All participants were members of an academy associated with a team competing in the top tier of Spanish soccer, La Liga. They were grouped according to their age category as U16 (n = 25; age = 15.8 ± 0.4 years; body height = 172.8 ± 5.6 cm; body mass = 64.7 ± 7.3 kg; body fat percentage = 10.5 ± 0.9%) and U19 groups (n = 35; age = 17.9 ± 0.8 years; body height = 174.3 ± 6.3 cm; body mass = 68.0 ± 5.9 kg; body fat percentage = 10.7 ± 1.1%). Goalkeepers were excluded from the data collection. The health condition of each player was validated by the club's medical personnel. Players who were injured during small-sided games (SSGs) or were engaged in rehabilitation training sessions were excluded from the data collection process. As the data used in this study were collected as part of routine player monitoring, informed consent was not considered necessary (Winter and Maughan, 2009). However, verbal consent was obtained from the club and the players, and anonymity was ensured. The study received approval from the Institutional Review Board of the University of Vigo (approval code: E1621871; approval date: 09 April 2021) and adhered to the principles outlined in the Declaration of Helsinki.

### Experimental Design

The present investigation was carried out during the competition period across two seasons (2020–2021 and 2021–2022). To achieve the study objectives, the relative and peak running demands of players during competition matches and SSGs were compared. U19 competition matches took place over a nine-month competition calendar period, starting from September and ending in May. On the other hand, U16 competition matches were played over a four-month competition calendar period, spanning from March to June. Data from each competition match were included only when players participated in the entire duration of the match. Similarly, for SSGs, files were incorporated into the analysis only if the player completed every SSG within the training session. The type of the SSG employed during each session was the choice of the coach and the conditioning coach. The players were well-acquainted with all SSG formats and task constraints, as these formats were regularly incorporated into training sessions by the coaching staff before the study commenced. Throughout the data collection period, there was considerable variation in environmental conditions, competition match schedules, and training times. During the season, U19 players were involved in 4–5 soccer-specific on-field training sessions and competed in one match per week. Conversely, U16 soccer players had three soccer-specific on-field sessions and also played one competition match per week. None of the analyzed teams changed coach during the study period (Marín and Castellano, 2023).

### External Load Variables

The locomotor variables collected from the GPS data were as follows: total distance covered (TDC), total distance covered relative (TDCR), high-speed distance (HSD) (>18–21 km•h^−1^), high-speed distance relative (HSDR), very high-speed distance (VHSD) (21–25 km•h^−1^), very high-speed distance relative (VHSDR), sprint distance (SD) (>25 km•h^−1^) and sprint distance relative (SDR). The total numbers of accelerations (TACC) and decelerations (TDEC) were also gathered, as well as their intensity (TACCR and TDECR, respectively). Total high accelerations (> 3 m•s^−2^) and decelerations (<−3 m•s^−2^) were also taken into account (THACC and THDEC) and relative to time (THACCR and THDECR, respectively) (de Dios-Álvarez et al., 2021b). Moreover, global indicators were included as variables, i.e., the number of efforts at high metabolic power (>20 W•kg^−1^) (HMP), and player load (PL) which is a variable based on the tri-axial accelerometer measures (Dalen et al., 2016). All normalized variables were assessed based on the following formula: SSGLoad = SSG external load/match external load. To determine the ApP that reproduces the normalized variables, TD, HSD, VHSD, SD, THACC, and THDEC were recorded during official matches across each age category.

### Training Content and Matches

A total of 978 (321 for U16 and 657 for U19) individual observations were undertaken (Table 1), with 15 different formats of SSGs included. For U16, SSGs ranged from 10 vs. 10 to 2 vs. 2 with an ApP from 60 m^2^ to 322 m^2^ (with 24 different ApPs). The SSGs’ duration in this category ranged from 1 to 31 min. For U19, SSGs ranged from 10 vs. 10 to 3 vs. 3 with an ApP from 54 m^2^ to 322 m^2^ (28 different ApPs), and the SSGs’ duration ranging from 2 to 32 min. The ApP was calculated excluding goalkeepers (Riboli et al., 2022) and was derived by dividing the overall pitch area by the total number of players present on the field (Castellano et al., 2015). Both small, medium, and large sided games were abbreviated as SSGs and specified by the ApP (ApP100: the ApP <150 m^2^•player^−1^; ApP200: the ApP ranged from 151 to 250 m^2^•player^−1^; ApP300: the ApP > 251 m^2^•player^−2^). Furthermore, the ApP was utilized as a continuous variable to examine its association with external load measures and to investigate its impact on GPS-derived external load metrics. Additionally, the ApP was treated as a discrete variable to compare the physical demands of SSGs with different ApP values against those observed in official matches. Throughout the SSGs, several coaches supervised and motivated players to maintain a high work ratio (Riboli et al., 2020). Moreover, whenever the ball went out of play, an immediate replacement ball was made available. In all SSGs, corners were replaced by using a ball in-game from the goalkeeper, except the 10 vs. 10 format, where corners were performed. Furthermore, to estimate external load measures during these tasks, the duration (in minutes) of SSGs was also taken into consideration.

**Table 1:** Description of the analyzed formats during the SSGs.

ApP	Format (# players per team)	Length per width (m)	Observations	Rules of play
ApP100	2 vs. 2	23 x 12	19	
		25 x 18	10	Goalkeeper and regular goals were used in all formats analyzed The number of ball contacts allowed was free No offside rule was applied The balls were replaced from goals The corners were replaced by a ball in game from the goalkeeper (except for 10 vs. 10 formats, where corners were performed). Verbal encouragement or feedback was allowed from coaches throughout the task. The neutral player (if there was) was always playing with the team in possession of the ball.
	3 vs. 3	18 x 22	38	
		28 x 18	24	
		32 x 22	15	
		32 x 30	16	
	3 vs. 3 + F	21 x 18	21	
	4 vs. 4	26 x 25	13	
		30 x 26	10	
		31 x 30	10	
		32 x 25	31	
	4 vs. 4 + F	32 x 24	22	
	5 vs. 5	26 x 23	20	
		27 x 25	54	
		30 x 26	24	
		30 x 32	6	
		32 x 25	31	
		35 x 28	22	
		32 x 32	7	
	6 vs. 6	35 x 32	44	
		38 x 30	18	
		36 x 36	10	
		38 x 36	12	
		45 x 34	12	
	6 vs. 6 + F	32 x 25	12	
	7 vs. 7	50 x 25	6	
		52 x 40	10	
	7 vs. 7 + 4F	50 x 40	11	
	8 vs. 8	45 x 52	6	
	10 vs. 10	45 x 52	14	
		52 x 62	39	
ApP200	8 vs. 8	54 x 50	6	
		60 x 42	5	
		62 x 55	30	
		61 x 62	14	
	10 vs. 10	58 x 64	28	
		60 x 62	24	
		70 x 52	5	
		70 x 56	6	
		62 x 65	7	
		67 x 62	42	
		70 x 62	5	
		72 x 64	7	
		75 x 62	28	
		76 x 62	13	
ApP300	8 vs. 8 + F	65 x 62	16	
		77 x 60	5	
	10 vs. 10	104 x 62	104	

A total of 62 and 53 individual observations of 10 official matches per age category were obtained for U16 and U19, respectively. Players were playing at U16 and U19 levels, which implies a normal size pitch (100 x 60 m, length and width, respectively) and the usual rules of 11-a-side soccer. Match activities were assessed from data collected only when players played the full match (Reynolds et al., 2021; Sal de Rellán-Guerra et al., 2019). Each match lasted for ninety minutes, divided into two halves of forty-five minutes each, with any extra time decided by the match referee. Uniform competition rules were followed for all matches, permitting each team to make three substitutions and allowing a fifteen-minute interval for half time. Prior to matches, a standardized twenty-minute warm-up routine was conducted, encompassing dynamic stretching, maximal sprint efforts of varying lengths, short and long passing drills, and SSGs (4 vs. 4 plus 2).

### Procedures

Participants undertook their traditional weekly training routine. All sessions were performed on artificial pitches and, all the games were scheduled at the same time of the day, for U16 between 19:30 and 20:30, while for U19 between 17:00 and 18:30. During both training sessions and matches, players' movements were captured using a portable 10-Hz GPS device, which also integrated a 400-Hz tri-axial accelerometer (Playertek, Dundalk, Ireland). Previous research (Scott et al., 2016) has indicated the validity and reliability of these GPS devices for team sports (CV = 1.9%, CV = 4.7 and CV = 10.5% for total distance, high-speed running and very high intensity running, respectively); additionally, they have been used in soccer research previously (de Dios-Álvarez et al., 2021a). To attach the GPS device securely, a special harness was used, and as per the manufacturers' instructions, all GPS units were activated 10 min before the training session or the match initiation. All external load measures were standardized as relative distance covered per minute (m•min^−1^) and meticulously integrated into the data analysis. Subsequently, to ascertain the ApP that would accurately replicate the normalized TD, HSD, VHSD, SD, and TACC/TDEC observed during official matches across each age category, these variables were recorded specifically during the official matches.

### Statistical Analysis

The results are presented as means and standard deviations (mean ± SD). Initially, all data were assessed for normality using the Shapiro-Wilk test. To investigate the relationship between the ApP and external load variables, Pearson correlation coefficients were employed. The magnitude of correlations was classified as trivial (r < 0.1), small (0.1 ≤ r < 0.3), moderate (0.3 ≤ r < 0.5), large (0.5 ≤ r < 0.7), very large (0.7 ≤ r < 0.9), almost perfect (r ≥ 0.9), and perfect (r = 1). Moreover, linear regression models were constructed to determine the potential of GPS-derived external load metrics to be predicted from the ApP. Furthermore, linear mixed models were performed to analyze the differences in external load variables according to the ApP (ApP100, ApP200, or ApP300). The players’ identity was modelled as a random effect to take into account the repeated measurements. The assumption of homogeneity and normal distribution was determined graphically for each model. All models presented a normal distribution and homogeneous variance. Effect sizes (ESs) were established using Cohen’s *d*. Concretely, ESs were calculated according to the formula *d* = (M2 – M1/SDpooled), where M1 and M2 were the means of the two groups and SDpooled was the square root of the weighted average SD for each SSG format (i.e., ApP100, ApP200, ApP300). According to Hopkins (2002), ESs were classified as trivial (< 0.1), small (0.1–0.3), moderate (0.3–0.5), large (0.5–0.7) and very large (>0.7). Data analysis was conducted using the statistical package SPSS version 25.0 for Mac (IBM. Co., New York, NY, USA) and R version 3.6.3 (R Core Team, 2018), with a significance value set at *p* < 0.05.

## Results

**Figure 1:**
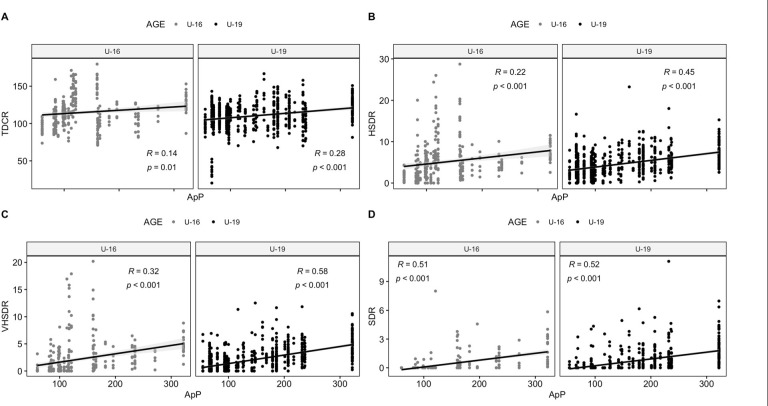
The relationship between external load measures and the area per player (m^2^•player^−1^) using small-sided games for different age categories is shown. *A: TDCR, total distance covered relative (m·min^−1^), B: HSDR, high-speed distance (>18–21 km·h^−1^) relative (m·min^−1^), C: VHSDR, very high-speed distance (21*–*25 km·h^−^^1^) relative (m·min^−1^), D: SDR, sprint distance (>25 km·h^−^^1^) relative (m·min^−1^)*

**Figure 2:**
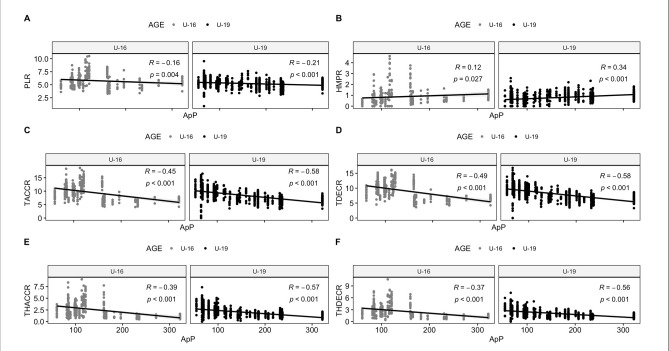
The relationship between external load measures and the area per player (m^2^•player^1^) using small-sided games for different age categories is shown. *A: PLR, player load relative (arbitrary units), B: HMPR, high metabolic power relative (>20 W·kg^−1^), C: TACCR, total accelerations relative (n·min^−1^), D: TDECR, total decelerations relative (n·min^−1^), E: THACC, total high accelerations (>3 m•s^−2^) relative (n·min^−1^), and F: THDEC, total high decelerations (<*−*3 m•s^−2^) relative (n·min^−1^)*

Table 2 presents the significant influence of the ApP on TDC, HSD, VHSD, and SD for U16 and U19 separately as well as combined. A positive effect was observed for the ApP on TDC (*p* < 0.001) when data from both U16 and U19 were combined. An increase of 10 m^2^ in the ApP and 1 min of play resulted in a corresponding increase of 116 m in TDC. Similarly, a positive relationship was found between the ApP and SD, with a 10-m^2^ increase in the ApP and 5 min of play resulting in an increase of approximately a 6-m rise in SD. Meanwhile, Table 3 showcases the best influence of the ApP and time of play on PL, HMP, THACC, and THDEC. When considering data from U16 and U19 together, an increase in the ApP had a positive effect on PL and HMP, but a negative impact on THACC and THDEC. In Figures 1 and 2, the relationship between the ApP and external load measures is depicted for each age group, considering both locomotor and mechanical values. Correlations for time motion measures ranged from r = 0.14 for TDC in U16 players to r = 0.58 for VHSD in U19 players. Regarding mechanical values, correlations ranged from r = 0.12 (HMP) in U16 players to r = −0.58 for U19 players in relation to TACC and TDEC.

**Table 2:** The influence of the area per player (ApP) and drill duration on TDC, HSD, VHSD and SD taking into account both U19 and U16 players separately and together (all).

Variables		ApP	Duration	Constant	R^2^	F
TDC (m)	U19	0.6***	114.0***	−101.2***	0.9	7136
	U16	3.2***	84.0***	−256.2***	0.9	3716
	All	1.6***	99.9**	−148.4***	0.9	9415
HSD (m)	U19	0.1***	6.2***	−20.1***	0.8	1052
	U16	0.4***	2.5***	−37.0***	0.7	478
	All	0.2***	4.4**	−25.5***	0.7	1365
	U19	0.1***	3.2***	−18.9***	0.7	826
VHSD (m)	U16	0.3***	1.2***	−29.9***	0.7	330
	All	0.2***	2.2**	−22.3***	0.7	1080
	U19	0.03**	1.6***	−9.4***	0.5	304
SD (m)	U16	0.1***	0.3*	−14.2***	0.4	105
	All	0.1***	0.9**	−10.9***	0.4	376

Note: * p < 0.05; ** p < 0.01; *** p < 0.001; ApP: Area per Player; TDC: total distance covered (m); HSD: high-speed distance (18–21 km•h^−1^) (m); VHSD: very high-speed distance (21–24 km•h^−1^) (m); SD: sprint distance (m) (>24 km•h^−1^). The duration variable was introduced in the model in minutes. For example, a total of 15 min of 5 vs. 5 (45 x 30 m) with an ApP of 135 m^2^•player^−1^ is equivalent to around 1500 m of TDC, 68 m of HSD, 36 m of VHSD and 13 m of SD.

**Table 3:** The influence of the area per player (ApP) and drill duration on PL, HMP, THACC and THDEC taking into account both U19 and U16 players separately and together.

Variables		ApP (m^2^)	Duration (min)	Constant	R^2^	F
PL (au)	U19	0.01	4.9***	0.4	0.9	6590
	U16	0.1***	3.9***	−5.4***	0.9	3338
	All	0.04***	4.5**	−1.2*	0.9	9210
HMP (W•kg^−1^)	U19	0.01***	0.9***	−1.9***	0.8	1253
	U16	0.04***	0.4***	−4.2***	0.8	541
	All	0.03***	0.7***	−2.7***	0.8	1597
	U19	0.02***	1.2***	6.4***	0.7	763
THACC (n)	U16	0.02***	1.0***	6.7***	0.7	371
	All	−0.01***	1.1**	6.2***	0.7	1089
	U19	0.02**	1.4***	6.1***	0.7	1006
THDEC (n)	U16	−0.01	1.1*	5.1***	0.8	5418
	All	−0.01***	1.3***	5.6***	0.7	1491

Note: * p < 0.05; ** p < 0.01; *** p< 0.001; PL: player load; HMP: high metabolic power (>25 W•kg^−1^); THACC: total high accelerations (>3 m•s^−2^); THDEC: total high decelerations (<–3 m•s^−2^). For example, a total of 15 min of 5 vs. 5 (45 x 30 m) with an ApP of 135 m^2^•player^−1^ is equivalent to around 21 THACC and 23 THDEC.

**Figure 3:**
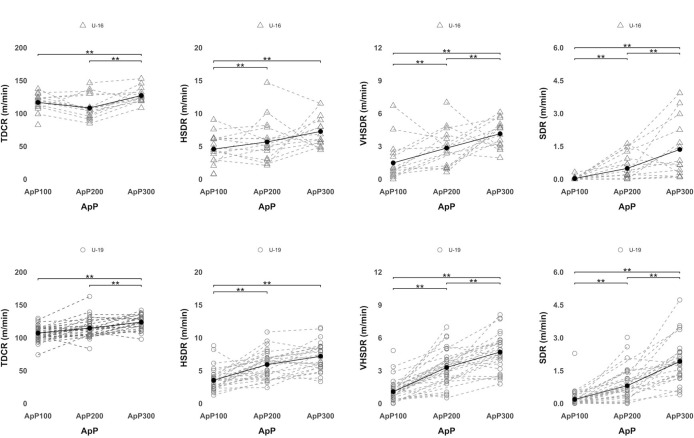
Locomotor external load differences among the different ApPs considering the average of each player. Triangles represent U16 soccer players, circles represent U19 soccer players. The black line shows the average taking into account all players. *Note: TDC, total distance covered relative (m·min^−1^), HSD, high-speed distance (>18–21 km·h^−1^) relative (m·min^−1^), VHSD, very high-speed distance (21*–*25 km·h^−^^1^) relative (m·min^−1^), SD, sprint distance (>25 km·h^−^^1^) relative (m·min^−1^)*.

**Figure 4:**
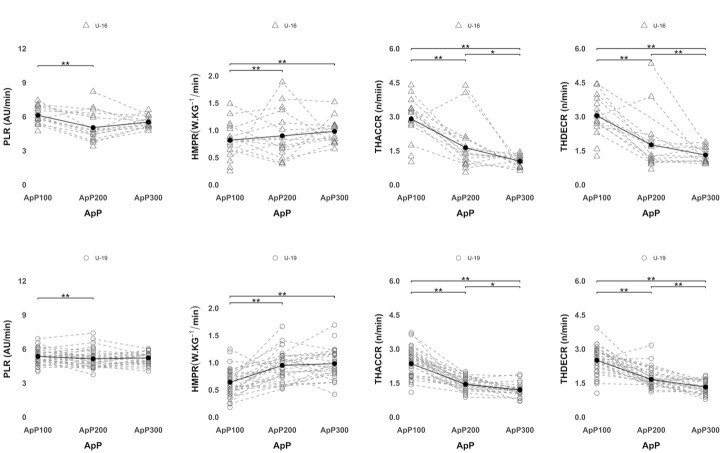
Mechanical external load differences among the different ApPs considering the average of each player. Triangles represent U16 soccer players, circles represent U19 soccer players. The black line shows the average taking into account all players. *Note: PLR, player load relative (arbitrary units), HMPR, high metabolic power relative (>20 W·kg^−1^), THACC, total high accelerations (>3 m•s^−2^) relative (n·min^−1^), and THDEC, total high decelerations (<*–*3 m•s^−2^) relative (n·min^−1^)*

Figure 3 shows differences among the three SSGs formats taking into account each player's average external locomotor load according to age category. Considering TDCR for both U16 and U19 soccer players, the ApP300 showed significantly (*p* < 0.0001) higher values than the ApP100 and the ApP200. Significantly (*p* < 0.0001) lower HSDR was covered during the ApP100 in comparison with the ApP200 and the ApP300 for U19 and U16 players. Regarding VHSDR and SDR, soccer players (U16 and U19) reported significantly (*p* < 0.0001) lower values in the ApP100 compared to the ApP200 and the ApP300. Also, players reached significantly (*p* < 0.0001) lower VHSDR and SDR values in the ApP200 compared to the ApP300 for both age groups. Figure 4 shows differences among SSG formats considering player's average external mechanical loads of each player according to the age category. Considering PLR, U16 and U19 soccer players showed significantly (*p* < 0.0001) lower values in the ApP200 than in the ApP100. For HMPR values, significantly (*p* < 0.0001) lower values were reported in the ApP100 compared to the ApP200 and the ApP300. Considering the number of THACCR, the ApP100 showed significantly (*p* < 0.0001) higher values than the ApP200 and the ApP300. Besides, the ApP200 showed a significantly (*p* < 0.05) higher number of THACCR compared to the ApP300 for U16 and U19 soccer players. The ApP100 reported significantly (*p* < 0.0001) greater THDECR values in comparison with the ApP200 and the ApP300. In addition, the ApP200 showed significantly (*p* < 0.0001) higher values than the ApP300 for both age groups.

**Figure 5:**
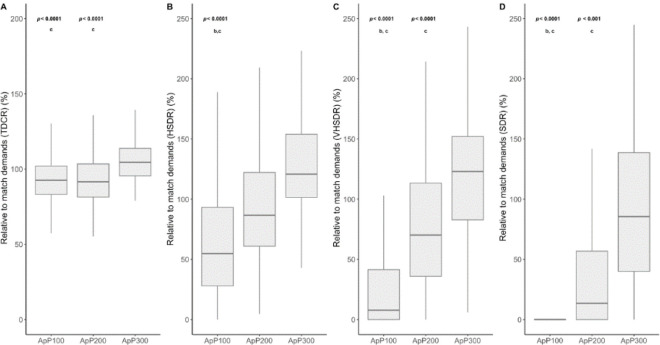
TDCR, total distance covered (A), HSDR, high-speed distance (>18–21 km·h^−1^) (B), VHSDR, very high-speed distance (21–25 km·h^−1^) (C) and SDR, sprint distance (>25 km·h^−1^) (D) relative to the match during small sided games using different areas per player (m^2^•player^−1^). *^b^ Significant differences with ApP200; ^c^ Significant differences with ApP300*.

**Figure 6:**
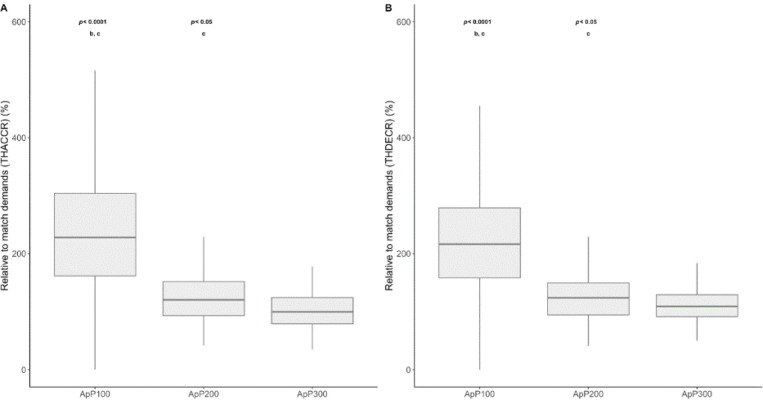
THACCR, total high accelerations (>3 m•s^−2^) (A) and THDECR total high decelerations (<–3 m•s^−2^) (B) relative to the match during small sided games using different areas per player (m^2^•player^−1^). *^b^ Significant differences with the ApP200; ^c^ Significant differences with the ApP300*.

Figures 5 and 6 present the comparison among different ApPs (ApP100; ApP200 and ApP300) for both locomotor and mechanical measures, taking into account the difference relative to average match demands. SSGs played with an ApP300 m^2^•player^−1^ showed significantly higher values in TDC (relative to match demands) compared to the ApP100 and the ApP200 (*p* < 0.0001; *d* = 0.67 and 0.66, respectively). Regarding HSD, the ApP100 showed lower values than the ApP200 and the ApP300 (*p* < 0.0001; *d* = 0.43, and 0.92, respectively). Significant differences were found among the ApP100, the ApP200 and the ApP300 in relation with match demands taking into account VHSD values. The ApP300 showed lower differences regarding match values in VHSD than the ApP100 and the ApP200 (*p* < 0.0001, *d* = 1.38 and 0.51). Moreover, the ApP200 exhibited significantly lower differences concerning match values compared to the ApP100 (*p* < 0.0001; ES: 0.7). Relative to match demands, the ApP100 reported higher difference in SD compared to the ApP200 (*p* < 0.0001; ES: 0.68) and the ApP300 (*p* < 0.0001; ES: 1.53). The ApP200 induced lower relative to match SD values in comparison with the ApP300 (*p* < 0.001; ES: 0.78). Concerning mechanical values, the ApP100 showed higher values compared to the ApP200 and the ApP300 in THACCR (*p* < 0.0001, ES: 1.0 and 1.5). The ApP200 resulted in higher THACCR values compared to the ApP300 (*p* < 0.05; ES: 0.53). In respect to THDECR, the ApP100 showed significantly higher values than the ApP200 and the ApP300 (*p* < 0.0001, ES: 0.68 and 1.53, respectively). The ApP200 showed higher THDECR values than the ApP300 (*p* < 0.05; ES: 0.78).

## Discussion

The aim of the study was to determine how the ApP influences physical responses measured by GPS-derived variables in youth soccer players and to compare physical demands of SSGs with different ApPs with those of official matches. The main findings were that moderate and large correlations were found between the ApP and locomotor measures for both U19 and U16 soccer players and large negative correlations were found between the ApP and mechanical measures for both age groups. The ApP300 showed greater mechanical (TDCR, HSDR, VHSDR and SDR) values compared to the ApP100 and the ApP200. However, SSGs played on the ApP100 showed the highest values considering mechanical (THACCR and THDECR) metrics. Additionally, SSGs played on less than the ApP300 underestimated the average locomotor activities (HSDR, VHSDR and SDR) reached during official matches. However, when mechanical effort was taken into account, all evaluated SSGs overestimated the average physical demands reported during official matches.

A higher ApP increased TDC, HSD, VHSD and SD. However, neuromuscular variables (i.e., PL, TACC, TDEC, THACC and THDEC) showed inverse correlation with the ApP for both U19 and U16 players. In line with the findings of previous research (Riboli et al., 2020; Sangnier et al., 2019), it

seems that a higher ApP increases time-motion measures, while as the ApP is reduced, higher neuromuscular values are showed. Indeed, as the ApP increases, players experience reduced ball possession, necessitating more running in defensive situations (to close gaps and apply pressure) and offensive scenarios (to open up spaces and build up play) (Owen et al., 2011). Nevertheless, our analysis showed that both positive and negative correlations were higher for U19 compared to U16 soccer players. A lesser physical development and a lesser understanding of the game may justify these findings. Furthermore, the age and expertise of players can significantly influence how they perceive and explore possibilities for themselves and their teammates within a specific context (Nunes et al., 2020). A greater experience causes greater synchronization and a better use of game information since youngest players tend to pursuit the ball and solve soccer-game problems by individual actions rather than seeking for a collective solution (Lemes et al., 2020). Consequently, it increases the relation and reduces the dispersion between the ApP and external load measures. This fact could explain the differences between both age categories.

The results have shown that it is possible to establish regression models that are representative of the external load measures with respect to the ApP during SSGs. For instance, based on our regression model, an increase of 10 m^2^ and 1 min of duration led to an increase of 116 m in TDC. Similarly, a positive effect was identified for the ApP and SD, where an increase of 10 m^2^ and 5 min of play corresponded to an increase of approximately 6 m in SD reached. Hence, this could help coaches and strength and conditioning specialists of youth soccer players use SSGs adequately. It is known that this type of drills induces positive effects on specific skills, decision-making, creativity, and sport-related physical fitness since they allow integrated training of technical, tactical and physical aspects (Sarmento et al., 2018). Therefore, conditioning through sport-specific drills represents a viable strategy to successfully mimic soccer-specific contextual elements.

Quantifying SSGs’ external load measures taking into consideration the ApP could be helpful in understanding the training process. Accordingly, it seems that an appropriate periodization of SSGs could effectively improve physical performance and prepare soccer players for official matches at both youth and senior levels (Clemente et al., 2014; Fernández-Espínola et al., 2020). The results of this study indicated that SSGs played on the ApP300 reported significantly higher values in HSDR, VHSDR and SDR. Our findings align with previous research, showing greater locomotor activities per minute in the ApP300 compared to the ApP100 and the ApP200 (Castillo et al., 2020a). On the contrary, the ApP100 showed higher mechanical values than other SSGs played on greater areas. Similarly, Clemente et al. (2019) indicated that large sided games (ApP300) had a lower number of accelerations compared to SSGs played on smaller areas (ApP100). However, when the number of players and the space are reduced during SSGs, frequent and short high-intensity actions both with and without the ball (e.g., dribbles, shots, and tackles) are increased (Clemente and Sarmento, 2020). Therefore, the observed increases in short high-intensity actions may account for the higher number of high acceleration and deceleration values recorded in the ApP100 when compared to those observed in the ApP200 and the ApP300.

The present study employed a novel approach to model the specific external load volume by including the ApP as a critical factor. The main findings were that, on the one hand, an ApP of less than 100 m^2^•player^−1^ did not stimulate enough HSD, VHSD and SD compared to official matches. However, this ApP overestimated neuromuscular variables such as THACC and THDEC. On the other hand, all ApPs analyzed reported similar values in TD to those reported in official matches. This similarity in TDC among all ApPs during SSGs and matches could be explained in part by the nature of this measure, which may simply not discriminate between both situations with the same sensitivity as other metrics do (i.e., VHSD, SD) (Reynolds et al., 2021). Hence, only SSGs played on the ApP >250 m^2^•player^−1^ reached similar VHSD and SD values to those found during official matches (Figure 5). To the best our knowledge, only Ribolli et al. (2022) investigated the relationships between the ApP and external loads to replicate official match demands in youth soccer players. The results of the present study are in accordance with those reported by the above-mentioned authors. In consequence, coaches and strength and conditioning professionals should take into account an approximate player-to-space ratio of 200 to 300 m^2^ per player to effectively mimic VHSD and SD activities during SSGs. This will ensure that the conditions, including the number of players, pitch size, and the nature of these actions (Oliva-Lozano et al., 2023) closely resemble those of a real match scenario.

To replicate SD demands during official matches, previous research carried out with French and Italian adult professional players used an ApP of approximately 311 and 316 m^2^•player^-1^, respectively (Lacome et al., 2017; Riboli et al., 2020). The findings in the current study are consistent with those observed in previous research. However, it seems that with young players a higher ApP during SSGs is needed to reproduce the competition demands. Apparently, young soccer players showed lower training and match intensity compared to adult professional soccer players (Reynolds et al., 2021), accordingly, adult players reached official matches demands with smaller ApPs. The variability in performance could be attributed to differences in biological maturation, enabling older players to better prepare for and consequently achieve more high-intensity efforts when SSGs are played on larger areas (Olthof et al., 2018; Rábano-Muñoz et al., 2019) obtaining a greater relationship between ApP and time motion measures. Taking into account neuromuscular values, 71 m^2^•player^−1^ were needed to reproduce these physical demands reported during official competitions. The result of the present study showed that SSGs played on the ApP <100 m^2^•player^−1^ imposed demands two to three times higher than those observed in an average match. This led to an overestimation of neuromuscular measures such as THDACC, THDEC and change of direction during training microcycles (de Dios-Álvarez et al., 2021a) and could lead to an increase in the incidence of injury.

However, some limitations should be mentioned with regard to the current investigation. Firstly, internal load measurements (i.e., the rating of perceived exertion and heart rate responses) were not analyzed and we should have considered these variables to describe accurately the physical demands. Secondly, both U16 and U19 soccer players did not play the same formats during SSGs. Thirdly, we did not consider playing positions and tactical behaviour, as previous research has found differences between positions (Lacome et al., 2017) and tactical formations (Asian-Clemente et al., 2022). This fact would be more remarkable when the ApP is increased (Martin-Garcia et al., 2019). Accordingly, an individualized approach for each position-game seems necessary. In addition, the players’ participation in official matches during the weeks of the study was not taken into account, which could influence the values reported in each of the SSGs analyzed. Lastly, a high number of training task observations used different areas per player. However, research considering more observations in the ApP200 and the ApP300 is needed to conclude more powerful outcomes.

Caution should therefore be exercised when interpreting these results. In addition, athletes’ characteristics, team playing styles and other contextual factors may have influenced the results. Increasing the number of categories studied may contribute to enhancing the comprehension of differences among various categories. Therefore, future studies should focus on further exploring the effects of the ApP, not only on physical demands but also on technical and tactical variables. Moreover, all SSGs analyzed were played without the offside rule and according to previous research (Castillo et al., 2020b), differences can be found when this constraint is taken into account. In consequence, future studies should investigate the ApP and external load measures using different training constraints that in the present research were not considered.

## Conclusions

The present study showed moderate to large correlations among the ApP and different GPS-derived external load measures for both U19 and U16 soccer players. The current findings reported that an ApP of less than 100 m^2^•player^−1^ in SSGs did not significantly stimulate HSD, VHSD and SD compared to official matches. However, the same ApP overestimated neuromuscular variables such as THACC and THDEC. Only SSGs played on an ApP > 250 m^2^•player^−1^ reached similar values to those found during official matches.

Our analysis revealed that both positive and negative correlations were greater for U19 in comparison with U16 soccer players. Perhaps the level of play and physical performance could explain the differences across both age groups.

The results demonstrated the feasibility of establishing regression models that accurately represented the relationship between external load measures and the ApP during SSGs. The outcomes could facilitate the effective modulation of training loads tailored to specific fitness components of players. Furthermore, a comprehensive calculation of the ApP is imperative to accurately estimate physical demands during SSGs and replicate the external load measures observed in official matches among elite youth soccer players. These insights have the potential to guide practitioners in recreating desired external load patterns aligned with official match demands by employing various ApP values within SSGs for elite youth soccer players.

## Practical Implications

The results of this study could help coaches and sport scientists anticipate external loads during SSGs. For example, it is feasible to see that a total of 15 min of 5 vs. 5 (45 x 30 m) with an ApP of 135 m^2^•player^−1^ is equivalent to around 1500 m of TDC, 68 m of HSD, 36 m of VHSD and 13 m of SD. Additionally, this task would report 21 THACCs and 23 THDECs. When strength and conditioning coaches for young soccer players aim to cover around 1500 m during SSGs, they should target an approximate playing area per player (ApP) of 200 m^2^•player^−1^ and play for 10 min. Hence, if they need to reach 150 m of high-speed distance (HSD), they could use SSGs with more than 200 m^2^•player^−1^ for approximately 20 to 30 min. In addition, if they want to adjust SD during this type of a soccer task to reach 50 m, they will need around 300 m^2^•player^−1^ and ~30 min of play. This evolution is consistent with findings reported by Casamichana and Castellano (2010) who observed a decrease in the distance covered to 125, 113, and 87 m•min^−1^ for ApP values of 273, 175 and 74 m^2^•player^−1^, respectively. Their results were highly comparable to those reported in the current study (128, 113 and 96 m•min^−1^, respectively) taking into account pooled data and SSGs with 10 min of duration. Indeed, conducting a thorough calculation of the ApP to estimate the physical demands during SSGs seems to be crucial to obtain external load measures similar to those observed during official games in elite youth soccer players. To develop similar demands of HSD, VHSD and SD in relation to official matches, it seems necessary to use an ApP >250 m^2^•player^−1^, which corresponds to a pitch size of 75 m in length by 53 m width, accommodating a total of 16 players on the field. This information could help select the most appropriate ApP for developing external load measures during SSGs with youth elite soccer players.
